# A skin-integrated device for neck posture monitoring and correction

**DOI:** 10.1038/s41378-023-00613-0

**Published:** 2023-11-28

**Authors:** Hu Luo, Tianhao Jin, Yu Zhang, Bohao Tian, Yuru Zhang, Dangxiao Wang

**Affiliations:** 1 State Key Lab of Virtual Reality Technology and Systems and Beijing Advanced Innovation Center for Biomedical Engineering, Beihang University, No. 37 Xueyuan Road, Haidian District, 100191 Beijing, China; 2 School of General Engineering, Beihang University, No. 37 Xueyuan Road, Haidian District, Beijing, China; 3 Peng Cheng Laboratory, Shenzhen, 518055 China

**Keywords:** Electrical and electronic engineering, Materials science

## Abstract

Cervical spondylosis is a common disease that is often caused by long-term abnormal cervical curvature due to activities such as reading books and using computers or smartphones. This paper explores building an untethered and skin-integrated device in an e-skin form factor to monitor and haptically correct neck posture. The proposed design features a multilayered structure that integrates all flexible electronic circuits and components into a compact skin space while being untethered and skin conformal. An accelerometer in the e-skin attaches to the neck for posture sensing, while four vibration actuators closely touch the neck skin to provide localized vibrotactile stimuli that encode four-direction correction cues of neck flexion $\pm \alpha$ and lateral bending $\pm \beta$. To ensure the reliability of posture sensing and vibrotactile rendering during neck movement, it is necessary to prevent the e-skin device from shifting position. Thus, a hollow structure-based method is implemented for stably attaching the e-skin to the neck skin. Experiments validated the e-skin device’s sensing precision, skin-conformal compliance, stickiness, stability and effectiveness during the motion of neck postures, including its discrimination of localized four-direction vibrotactile cues. A user study verified the device’s performance for sensing and correcting different abnormal neck postures during activities such as using smartphones, reading books, and processing computer files. The proposed e-skin device may create opportunities for more convenient cervical spondylosis prevention and rehabilitation.

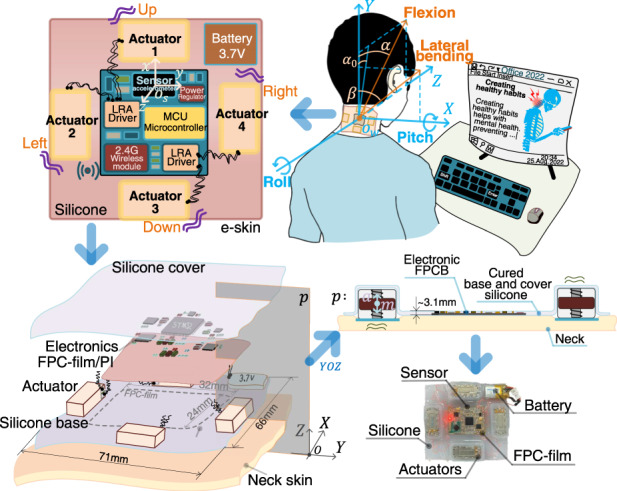

## Introduction

Due to changes in lifestyle and work, many jobs and forms of entertainment lead people to maintain a fixed posture of the head and neck for extended periods, such as when using computers, smartphones, or tablets^1–7^. As a result, prolonged abnormal neck posture can lead to irreversible pathological changes in the cervical spine, known as cervical spondylosis, which is a chronic degenerative process^8–11^. This condition can eventually result in symptoms such as numbness, weakness, tingling in the neck and/or arms, pain in the neck, neck stiffness, and headaches^12^. As reported in ref. ^13^, neck pain had an age-standardized prevalence rate of 27.0 per 1000 people worldwide in 2019.

Studies in ref. ^1^ further proposed that prolonged abnormal neck posture is detrimental to the cervical discs of the cervical vertebrae, leading to cervical disc degeneration. A study^7^ reported that one mechanism of the development of cervical spondylosis is increased cervical flexion for long hours, which results in cervical instability due to progressive wear and tear changes in the cervical spine that progressively degenerate.

Fortunately, clinical practice has shown that early abnormal cervical curvature can be corrected through cervical spine posture monitoring and therapeutic intervention, with the process being more effective the earlier it begins^14–16^. However, current efforts to address this issue typically rely on either 1) expensive motion capture systems in a laboratory setting or 2) rigid and discrete devices that trigger audio-visual feedback on a smartphone or computer, which may not be practical or convenient for users to monitor and prevent cervical curvature abnormalities^16–27^.

Motion capture systems have typically been used for neck posture detection due to their relatively high accuracy and reliability^17–19,25^. For example, Haruhi et al.^18^ developed a method for establishing pure cervical range of motion measurements using a three-dimensional motion analysis system (VICON). However, this work focused on monitoring neck posture instead of correcting cervical curvature abnormalities. Similarly, in ref. ^25^, an OptiTrack camera system was used to monitor user neck posture while they were using smartphones. Yet optoelectronic motion capture systems are expensive and require specialized laboratory settings, making them less convenient and affordable for collecting data in the field under natural circumstances.

Therefore, prior research has also explored using magnetic and inertial measurement units such as accelerometers, gyroscopes, or magnetometers to monitor neck posture^20–24,28–30^. Breen et al.^28^ built a system by placing a single accelerometer module at the C7 vertebrae for neck posture monitoring and correction. The correction strategy for user neck posture adopted both visual and auditory feedback when users were sitting in front of a computer screen. In contrast, Yingying et al.^14^ developed a detecting algorithm for classifying seven different neck postures and recording the duration of a user’s fixed abnormal cervical curvature using an accelerometer module wrapped in a headband and centered around the user’s forehead. A smartphone application performed data collection and sent data to physicians for online monitoring and diagnosis. Jasiewicz et al.^21^ verified the performance of using a rigid orientation-sensor embedded box (called IC3, integrating an accelerometer, a gyroscope, and a magnetometer) to measure neck movement range, seeking to widen the possibilities for developing new quantitative assessment techniques for neck diagnosis and treatment. In addition, an accelerometer-based method was further shown to be simple and effective for measuring head excursion and neck postures in ref. ^22^. The accelerometer was shaped as an integrated flat box secured on the participant’s forehead with a headband, quantifying head excursion among neck pain patients.

Distinct from previous methods, in which the IMU sensor was placed on the neck or forehead, Jeong et al.^23^ attached accelerometer modules to one ear. Their work studied a model-fitting algorithm for monitoring neck postures and providing exercise suggestions for neck pain prevention. Focusing on neck posture monitoring, other schemes, such as using optical fiber sensors^31,32^, elastic-material-based flex sensors^33–35^, or wearable speakers based on ultrasound pressure^36^, have also been used for neck posture monitoring. However, these systems might be impractical for widespread use due to the high cost of flex sensors and ultrasound hardware and the inconvenient form factors of the resulting devices as compared to an intact and untethered e-skin device.

Regarding smartphone usage scenarios, attempts have been made to use smartphone built-in sensors for neck posture monitoring and alerting^26,27,37–39^. Lawanont et al.^37^ proposed algorithms to monitor, alert, and summarize the neck angle of the user while using a smartphone based on a combination of the smartphone’s built-in accelerometer, magnetic sensor, and image detection techniques (face feature detection by phone front-facing camera). The method provided alerts to the user if their face position was unhealthy for a certain period. More recently, Gupta^27^ proposed a posture-detecting mechanism that directly converted data from the built-in triaxial accelerometer into useful values analyzed for monitoring user neck postures. Then, visual notifications of the posture severity were provided to users when it was determined to be incorrect. The visual prompts were removed automatically when the abnormal posture was corrected.

Instead of using discrete rigid components and sending the sensed data to physicians for assistance or alerting users for correction by visual and auditory feedback via mobile phones or computers, this study aimed to investigate whether an untethered device in the e-skin form factor could be built to stably sense and correct human neck postures using one inertial sensor and four vibrotactile actuators.

**Fig. 1:**
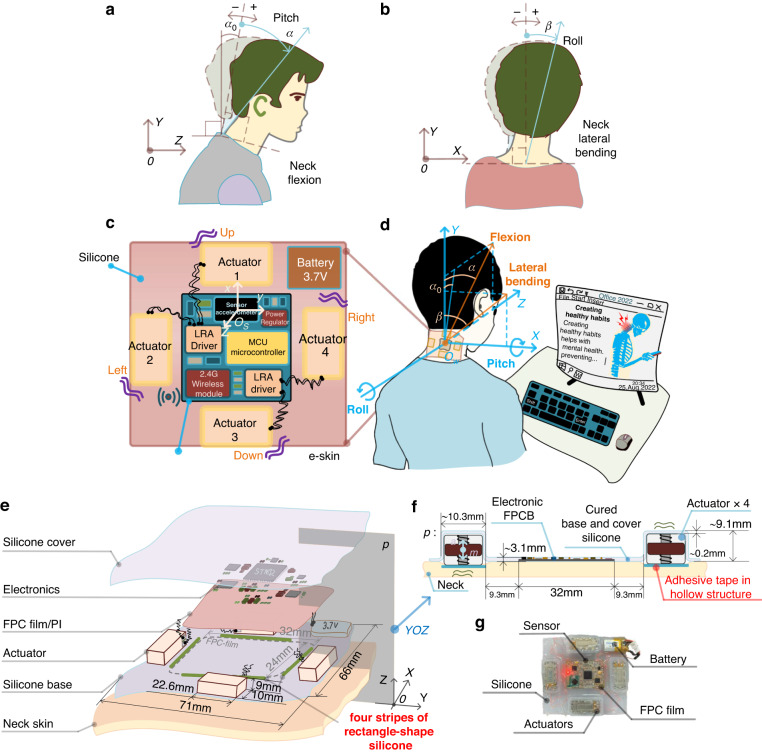
User neck posture angle of pitch α and roll β, and architecture, structures, user scene, and design parameters for manufacture of the e-skin device. **a** Neck angle of pitch $\alpha$ is defined by the neck flexion, and ${\alpha }_{0}$ is the natural physiological neck flexion angle when users are in healthy posture. **b** neck angle of roll $\beta$ is defined by the neck lateral bending. **c** 2D ($x$-$y$) illustrations of the silicone-based e-skin which encapsulates the flexible printed circuits, sensor, and four actuators. **d** e-skin is attached to user’s neck, and she is dealing with office work with her neck inclining a flexion pitch angle $\alpha$ and bending a lateral roll angle $\beta$. **e** This shows the vertically casting process, assemble relationships, and details of the silicone encapsulated e-skin. **f** This shows the assembled and cured silicone e-skin cross-sectioned by $p$ in plane ${YOZ}$, and the wavy lines “~” in front of parameters indicate rough sizes due to the silicone casting process. **g** This shows the made e-skin prototype in which the silicone-encapsulated electronic parts are surrounded by four direction-coding actuators

According to a medical assessment of the stresses on the cervical spine caused by posture and head position^16^, the most efficient position for the neck is when the ears are aligned with the shoulders. Such proper alignment can reduce spinal stress on the neck. Therefore, as shown in Fig. 1a, b, the presently built e-skin device, which uses one accelerometer sensor and four localized direction-coding vibration actuators, is designed to monitor and correct neck posture for a normal state of healthy posture in terms of four angle directions: the pitch $\pm \alpha$ of neck flexion and the roll $\pm \beta$ of lateral bending. The healthy posture is depicted using dashed lines in Fig. 1a, b. The pitch angle ${\alpha }_{0}$ represents the natural physiological neck flexion angle when users are in a healthy posture.

An inertial measurement unit (IMU)-based sensor in the e-skin device is position-fixed with the neck for posture sensing, and vibration actuators are required to closely touch the neck skin and provide vibrotactile direction-coding correction signals. However, during neck movement, satisfying these two requirements for an untethered e-skin device can be challenging. Using a silicone-based e-skin that has low adhesion to attach to the neck can result in position shifts during neck flexion and lateral bending, leading to posture sensing and vibrotactile rendering failures. Additionally, using a tightening bandage around the neck to fix the e-skin can cause inconvenience and discomfort for the user and may not prevent the e-skin device from shifting position along the neck.

The technical contributions of this paper are twofold. First, a multilayered structure design is proposed to integrate all flexible electronic circuits and components into a compact skin space to create a form factor for the e-skin device. This design scheme ensures that the e-skin device is untethered, compliant, and skin-conformal when attached to the neck. Second, we propose a hollow-structure-based method (an attaching scheme) for robustly attaching e-skin to neck skin. This method can prevent the e-skin device from shifting position, ensuring the reliability of posture sensing and localized vibrotactile rendering during neck flexion and lateral bending.

Thus, based on the design of the e-skin with an appropriate form factor, evaluation tests were performed to validate the sensing precision, mechanical properties for skin-conformal compliance, stickiness, effectiveness for following the motion of neck flexion and lateral bending, and the perceptual isolation of localized vibrotactile discrimination. Additionally, user studies were further designed to verify the performance of the e-skin device in neck posture sensing and abnormal neck posture correction in typical situations of using smartphones, reading books, and processing office files on computers.

To the best of our knowledge, there have not yet been any reported studies on a silicone-based device in an e-skin form factor that integrates both sensing and vibrotactile stimuli to monitor and correct user neck postures for cervical spondylosis prevention. The proposed e-skin device may, therefore create opportunities for cervical spondylosis prevention and rehabilitation.

## Results

### E-skin design analysis

The e-skin device can have a rectangular, rhombic, or rounded shape. To measure neck posture, the IMU-based e-skin must be positioned and oriented in alignment with the axis line of the cervical spine and the horizontal line of the shoulders. However, the invisible diagonal reference lines of a rhombic shape and the lack of reference lines in a round shape make positioning and orienting challenging. Therefore, we adopted a rectangular outline for the e-skin shape.

Next, we measured the length and width of the hairless skin on the back of the neck in six subjects (mean body weight: 78.3 ± 25.5 kg). The length was defined as the distance between the horizontal line of the shoulders and the end of the hair on the head, and the width was defined as the distance between the ends of hair on the left and right sides of the rear neck skin. Based on these measurements, a mean size of 66 mm × 71 mm was used for the e-skin.

The IMU-based sensor in the e-skin device needs to be fixed in position with the neck for posture sensing, and the vibration actuators need to closely touch the neck skin to provide vibrotactile correction signals. However, satisfying both of these conditions while the neck is in motion is challenging for an untethered form factor of the e-skin device. Using a tightening bandage around the neck to fix the e-skin can cause user discomfort and cannot prevent the e-skin from shifting position along the neck, leading to failed posture sensing and vibrotactile rendering during neck flexion and lateral bending. The e-skin needs to stably and closely attach to the neck skin to follow neck motion. To achieve this goal, two priorities are emphasized: 1) ensuring adequate overall mechanical properties of the e-skin, including flexibility, bending, and twisting for compliance and skin-conformal; and 2) ensuring sufficient stickiness between the e-skin and the neck skin.

Regarding priority 1, after integrating all electronic components, including sensors, drivers, actuators, controllers, wireless communication modules, and power units, into a compact skin space, the silicone-based e-skin device must ensure overall skin conformal compliance. To secure this piece, we chose commercial electronic chips and components as small as possible, given that e-skin with small thickness and low density layouts of flexible print circuits (FPC) would contribute to the mechanical properties and localized vibrotactile stimuli rendering. We then optimized the layouts of the chips and serpentine connecting wires on the FPC film, resulting in a size of 32 mm × 24 mm. Four vibration acturators for direction corrections ($\pm \alpha ,\pm \beta$) were placed surrounding the four sides of the FPC film, keeping a distance as far as possible to avoid mutual vibration interference between the actuators and the FPC film, as shown in Fig. 1c.

To encapsulate all parts and components into an e-skin form factor, we used a silicone casting process for encapsulation. However, the thickness sizes of chips and components varied, and a unified e-skin thickness size depended on the thickest component, which in our design was the vibration actuator. This added extra weight to the e-skin device and could damage both the mechanical properties and the localized vibrotactile stimuli rendering. To address this, we adopted a multilayer casting method, which cast silicone encapsulation layers according to the significantly different thickness sizes of the components, as shown in Fig. 1e, f.

For consistency between e-skin devices, by using a designed 3D printed mold with four rectangular groove holes, we cast four stripes of rectangle-shaped silicone on the e-skin silicone base layer (as shown in Fig. 1e). Next, we put the rectangular electronic FPCB into the surrounding area of the four stripes of silicone (while the IMU accelerometer sensor is designed at the middle of the FPCB, see Fig. 1c). The final casting of silicone cover encapsulates the silicone base and the four stripes of silicone together. In this way, the consistency between devices can be guaranteed. For more details on the encapsulation and fabrication process, please refer to supplementary material S1.

Regarding priority 2, the stickiness scheme for stable attachment of the e-skin mounting on the neck can be crucial during repeated use and neck motion. The silicone-encapsulated e-skin is originally sticky during attachment, but this stickiness degrades over time due to accumulated dust and user perspiration. The resulting low stickiness can lead to position shifts of the e-skin during neck movement and can ultimately cause failure in neck posture sensing and vibrotactile rendering.

To solve this problem, we use medical adhesive double-sided tapes with strong and skin-friendly stickiness; most importantly, it is also replaceable. However, we found that the tapes did not work when attempting to stick to the bottom silicone surface of the e-skin. To address this issue, especially in the bottom e-skin silicone, we designed a hollow structure that exposed the bottom metal surface of cuboid vibration actuators (the remaining five surfaces were encapsulated by silicone). This modification allowed the tapes to stick tightly to the metal surface of the actuators. In the end, we applied replaceable medical adhesive double-sided tape onto the exposed metal surface of the actuators in the hollow structure (see Fig. 1f) and attached the e-skin to the neck (see Fig. 1d, g).

### Sensing test

A digital level was used as a criterion to validate the sensing ability of the e-skin device. The digital level (GIM60 Professional, BOSCH, Germany) is intended for precise measuring of inclines. Its accuracy for $0^\circ$ or $90^\circ$ is $\pm 0.05^\circ$, and for $-1^\circ -89^\circ$, it is $\pm 0.2^\circ$.

**Fig. 2:**
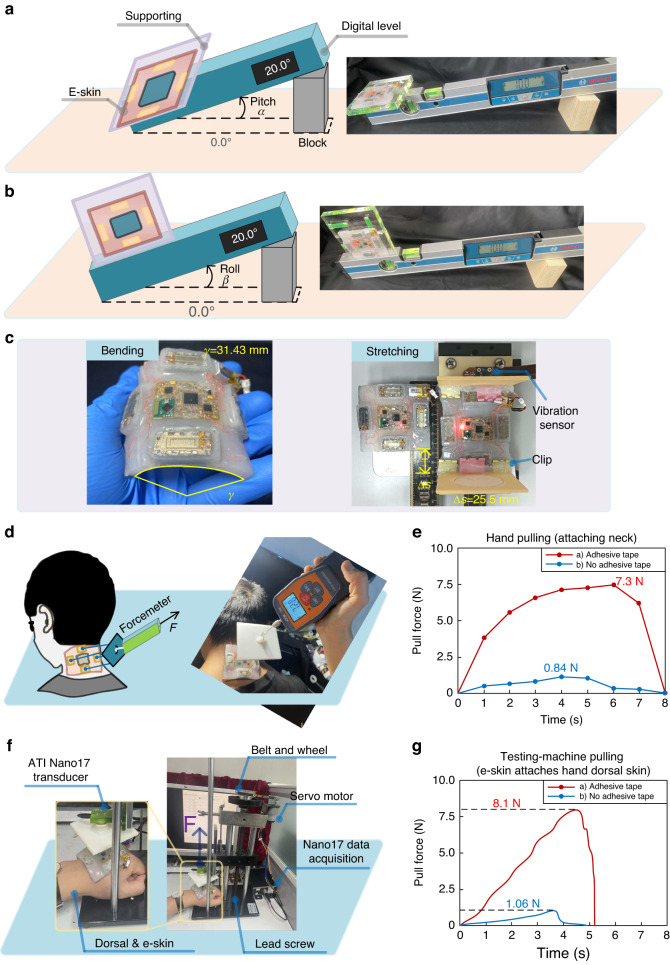
Validation of angle sensing, mechanical property and stickiness, and vibrotactile stimuli localization. **a**, **b** These show using a digital level in two ways for scaling the e-skin pitch and roll. **c** It shows the bending and stretching test of the e-skin device. **d** It shows using a digital forcemeter to scale the stickiness of the neck attached e-skin. **e** This shows the results of our attaching scheme of hollow-structure based adhesive-tape and directly attaching without adhesive-tape. **f** It shows using an ATI force sensor system to measure the e-skin adhesion on a hand dorsal. **g** It shows the results of e-skin adhesion on a hand dorsal

The setup for testing the angle pitch and roll of the e-skin is shown in Fig. 2a, b. The e-skin is attached to a supporting board. The board is sticked and aligned with the digital level in two ways for scaling the pitch and roll, respectively. We began testing with the digital level reading 0°. Next, the digital level was rotated with an increment $\Delta \alpha$ $=$ 10° and $\Delta \beta =$ 10° by moving a block, resulting in 18 increment angle positions for both pitch and roll. Throughout this process, the vibration strategy was designed. For each increment angle position, we read and averaged five sensed values from the e-skin.

The root mean squared error $\sqrt{\sum {({eskin}(\theta )-{level}(\theta ))}^{2}/18}$ for $\theta =\alpha$, $\beta$, is ${\alpha }_{{RMSE}}=1.37^\circ$ and ${\beta }_{{RMSE}}=1.14^\circ$, respectively. Therefore, we can conclude that the filtering algorithm and neck posture estimation process are sufficiently accurate in rendering vibrotactile stimuli. The e-skin device is able to measure the angle of pitch and roll with this precision.

**Fig. 3:**
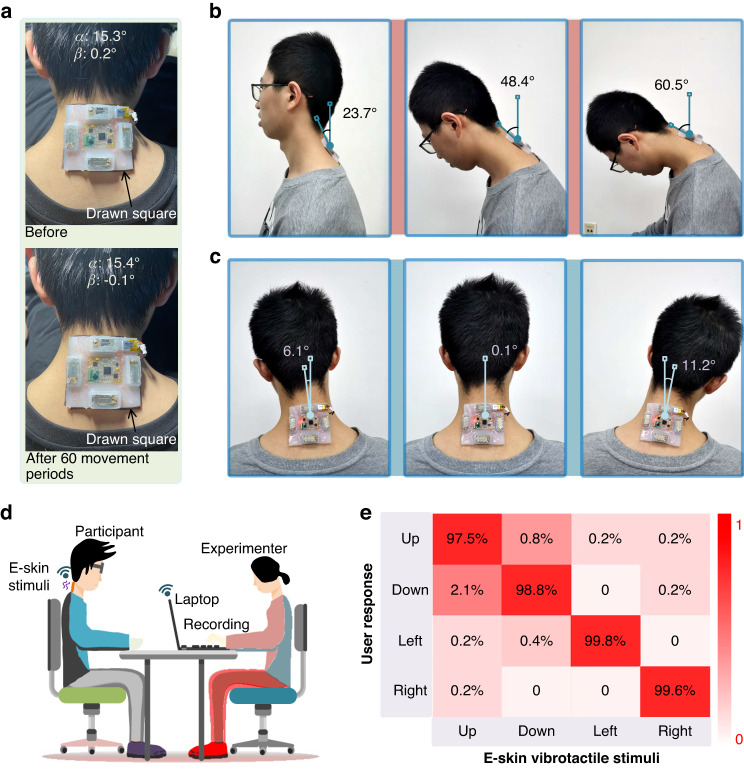
E-skin’s stability and stickiness in motion of neck, and the correct rate of discriminating localized vibrotactile stimuli. **a** This shows the stability of stickiness before and after repeated times of continuous neck movement by a drawn square. **b**, **c** These show the typical neck postures of flexion and lateral bending measured by e-skin. **d** It shows the test design of discriminating vibrotactile stimuli. **e** This shows correct rate confusion matrix of the discrimination in 480 trials at the four locations

As shown in Fig. 3b, c, the e-skin measures users’ neck posture to a certain extent, with flexion at 60.5° and lateral bending at 11.2°. In this condition, the accuracy of ${{{\alpha }}}_{{{RMSE}}}={{1.37}}^\circ$ and ${{{\beta }}}_{{{RMSE}}}={{1.14}}^\circ$ accounted for 2.26% (i.e., ${{1.37}}^\circ$/60.5°) and 5.09% (i.e., ${{1.14}}^\circ$/(|$\pm$11.2° | ×2), $\pm$11.2° is because for lateral bending, the movement could be bidirectional), respectively.

In our user study, the angular intervals of each step were set to 15° for flexion and 5° for lateral bending. The results show that participants triggered neck flexion abnormalities much more frequently (121 times), even in a 15° step, compared to neck lateral bending in a 5° step (24 times). This indicates that the frequency of neck flexion abnormalities is at least five times higher than that of lateral bending.

Additionally, our main objective is to sense users’ neck posture and correct abnormities to the normal range (i.e., 0–15° for flexion and −5°~ + 5° for lateral bending), instead of maintaining a precise goal posture.

Thus, considering these three aforementioned reasons, we propose that the accuracy of ${\alpha }_{{RMSE}}=1.37^\circ$ and ${\beta }_{{RMSE}}=1.14^\circ$ for e-skin devices is adequate for performing neck posture sensing and correction.

### Mechanical properties and stickiness

We tested the e-skin’s mechanical properties by bending and stretching the device, as shown in Fig. 2c, and validated its skin-confomal compliance by attaching it to a human neck, as shown in Fig. 3b, c.

For neck lateral bending, the e-skin device needs to be compliant over the direction of the neck circumference. Therefore, e-skin devices need to have a radius of curvature larger than the human neck and should be able to stick to the neck over the neck circumference direction. As shown in Fig. 2c (left), the radius of curvature of e-skin can be 31.43 mm→+∞ mm (+∞ mm means a flat placed e-skin), which includes a human neck equivalent radius of curvature of 50.92 mm [40].

For neck flexion, although we did not find skin stretch rate data exactly on the neck, we obtained a forearm skin stretch rate of up to 30%^41^. Furthermore, we measured the neck skin stretch rate in three participants who performed neck flexion to their maximum, obtaining a mean value of 22.7%. Next, we used a test mechanical system to measure the stretch rate of the encapsulated e-skin device, as shown in Fig. 2c (right), and at least a 38.6% stretch rate [i.e., (91.5 − 66)/66 mm] was obtained. We note that the silicone (Ecoflex^TM^ 00-33) used in this study can be stretched >900%.

After the bending and stretching tests, the functioning of e-skin vibration is validated by using an accelerator board from TI (ADXL326, DRV-AAC16-EVM, TI). Next, the sensing accuracy is measured using a digital level (GIM60 Professional, BOSCH, Germany) by the same process as described above, and the accuracy is ${\alpha }_{{RMSE}}=1.45^\circ$ and ${\beta }_{{RMSE}}=1.18^\circ$.

To measure the stickiness when attaching on the neck, we used a digital forcemeter (precision: $\pm$ 0.5%, VC10N, Victor Instrument Co. Ltd., China) with two different attachment schemes: a) hollow-structure-based adhesive tape and b) direct attachment to the neck, as shown in Fig. 2d. Four strings were used to link four actuators to a board using hot melt glue. We then pulled the forcemeter until the e-skin was removed from the neck skin, recording force data throughout the process, as shown in Fig. 2e. Our attachment scheme using hollow-structure-based adhesive tape improved the stickiness force to 7.3 N from 0.84 N when the e-skin was directly attached without adhesive tape.

To further quantify e-skin adhesion, we used a universal testing machine to measure the pulling force of e-skin adhesion. The testing machine system consists of an ATI Nano17 force sensor (ATI Industrial Automation, USA) and a pulling mechanism (i.e., a controlled servo motor with a belt and wheels for driving a lead screw), as shown in Fig. 2f. The bottom side of the e-skin device attaches to the dorsal skin of a fisted hand, and the upper side of the e-skin is four-string-connected with a square board that is fixed with the Nano17 force transducer.

An Arduino UNO board controled the servo motor to move the Nano17 force transducer up along the lead rail until the e-skin device is fully split up. During the process, the transducer measures the pulling force (*F*) at a rate of 1 kHz. Thus, the maximal adhesive force in the two conditions of adhesive tape (i.e., 8.1 N) and no adhesive tape (i.e., 1.06 N) is shown in Fig. 2g (the gravity 0.64 N of the e-skin and the square pulling board is subtracted).

Because people typically work or study for a whole morning (9:00–12:00) and/or a whole afternoon (14:00–17:00) (assumming breaks for eating and resting at noon), we attached an e-skin device to a participant for an entire day excluding the noon break. During the noon break and after completing the test, we measured adhesive force data using the universal testing machine as mentioned. The results show that the pulling force is degenerating [8.1 N (new medical double side tape) ${\boldsymbol{\to }}$ 6.3 N (noon break) ${\boldsymbol{\to }}$ 4.1 N (test complete)]. Upon analyzing the underlying reasons for this data, we believe that during the process of detachment and attachment to the neck and hand-dorsal (testing machine), it cannot prevent the e-skin device from repeated dust/perspiration accumulation and loss of adhesive material.

To further validate the effectiveness and stability of the stickiness during neck motion, a participant was asked to perform 60 repeated continuous movements of neck flexion from 0° to 60.5° and lateral bending from −11°–11° over ~15 min. As shown in Fig. 3a, no observable displacement was observed when comparing the drawn square before and after 60 movement periods. Additionally, as shown in Fig. 3b, c, typical neck postures could be measured by the e-skin. Therefore, we can conclude that e-skin can stably attach to the participant’s neck and follow changes in different neck postures during flexion and lateral bending.

### Localized vibrotactile stimuli

To evaluate the e-skin’s performance for independently rendering vibrotactile stimuli to participants on the neck, twelve graduate students (five females) aged 22–28 were recruited from Beihang University.

As shown in Fig. 3d, participants sat up straight by mounting the shoulder-aligned e-skin, and the test was introduced to them. The experimenter used a laptop with Bluetooth to command the e-skin to render four vibrotactile stimuli in the order of actuator 1 (up), 3 (down), 2 (left), and 4 (right). Each localized stimulus lasted for 2 seconds and was followed by a 2-second pause. This sequence order of rendering was repeated a total of ten times. Then, the formal test began. Headphones playing pink noise were used to block faint sounds produced by the actuators.

Each formal trial consisted of a sequence of 2 s vibrotactile stimuli and a 2 s pause by a randomly selected actuator out of the four. During the 2 s pause, participants vocally reported the sensed stimulus location as “Up, Down, Left, or Right.” Answers were recorded after each report. Each actuator rendered stimuli a total of 40 times, resulting in 160 trials for each participant and costing ~15 min for the whole test.

The correct rate of discriminating the four localized stimuli was used for objective evaluation and is shown in Fig. 3e. The correct rate for each location was more than 97.5% in 480 trials (average of all four locations: 98.9%). Thus, we conclude that the e-skin device in this form factor design is able to render localized vibrotactile stimuli that can be discriminated by participants (perceptual isolation). Furthermore, this localization ability is proposed as being useful for correcting abnormal neck postures.

### User study

**Fig. 4:**
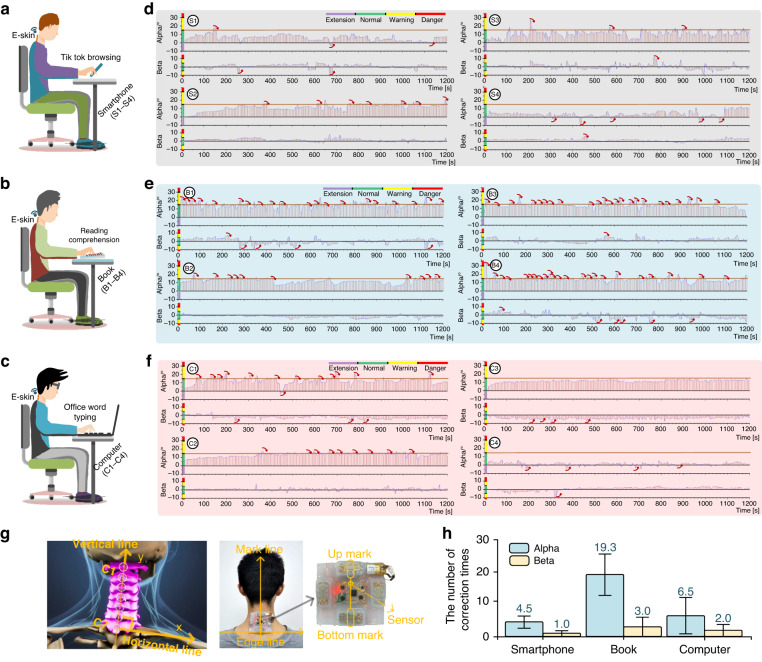
User study results of three scenarios. **a**–**c** These show three scenarios. **d**–**f** It show the recorded temporal neck posture and the triggered events of vibrotactile correction by e-skin in scenario of using smartphones, reading books, and using computers. **g** It shows the placement of e-skin device. **h** This shows the mean number of correction times of the triggered events. Error bars indicate standard deviation

We sought to monitor the posture of the neck cervical spine, so it is logical to place the posture sensor of the e-skin device as close to the cervical vertebrae as possible regarding the directions of horizontal *x* and vertical *y*. Cervical flexion or lateral bending is a complex mechanism involving eight joints (i.e., the skull and seven cervical vertebrae C1–C7)^28,42^, as shown in Fig. 4g (left). C7 is the base of neck movement, and the final posture of the neck is an accumulated result of posture changes from C1 to C7.

Therefore, as shown in Fig. 4g (right), for the vertical direction *y*, we placed and aligned the bottom edge line of the e-skin device with the horizonal line of two shoulders that passed vertebrae C7. For the horizonal direction *x*, we placed the e-skin mark line (i.e., point up-mark and point bottom-mark, and the middle points of the two marks are guaranteed by the casting mold and the actuators) to align with the central vertical line that passes C1–C7.

The user study sought to validate the practical effects of the e-skin device in three typical scenarios of entertainment and work, as shown in Fig. 4a–c: playing smartphones, reading books, and dealing with files on computers. To unify the experimental conditions for attaching the e-skin device for the user study, participants conducted a zero-calibration process to initiate angle ${\alpha }_{0}$ in Eq. (1). These participants sat up straight with their eyes looking ahead and both ears aligned with their shoulders [16]. Then, the e-skin was attached to the neck. This sensed neck posture data in the e-skin was initialized and regarded as the zero position.

The same group of twelve participants also participated in the user study and were randomly divided into three groups for the three scenarios. Each scenario consisted of four participants, i.e., S1–S4 for smartphone playing, B1–B4 for book reading, and C1–C4 for computer use. They were introduced to the three scenarios and the vibrotactile correction strategies before using the e-skin device.

Throughout the test, participants were asked to focus on their tasks until the end: 1) browsing the Tik Tok app on smartphones; 2) reading selected passages of a book and answering several reading-comprehension questions by filling in the blanks on a piece of A4 paper; and 3) copy-typing words from a PDF file to a Word file in the Microsoft Office app on a computer. Each scenario lasted for 20 min for each participant. We designed the workloads of the tasks to be far more than 20 min. Participants stopped only when they were told that the time was over. Headphones playing pink noise were used to block faint sounds that might be produced by the actuators.

During the formal test, the e-skin monitored the neck posture of the participants in real time and wirelessly uploaded the sensed pitch and roll ${\left[\alpha ,\beta \right]}^{T}$ data to a laptop at 5-s intervals. Meanwhile, once the neck posture met abnormal conditions of (3) and/or (4), the e-skin uploaded then timestamp ${t}_{c}$ and posture data ${\left[{\alpha }_{c},{\beta }_{c}\right]}^{T}$ to a laptop and performed the vibrotactile correction using strategy (5). When participants entered the normal posture state, another timestamp ${t}_{s}$ was recorded.

### Analysis

As shown in Fig. 4, temporal neck posture data ${\left[\alpha ,\beta \right]}^{T}$ and the triggered events of vibrotactile correction by the e-skin in the scenarios of using smartphones, reading books, and using computers can be seen in Fig. 4d, e, and f, respectively. Red arrows are depicted in temporal plots when vibrotactile correction events occur. Regarding the different states of participants’ neck postures, the states of extension, normal, warning, and danger are marked in different colors. As observed in the three scenarios, the proposed e-skin device can monitor the neck posture pitch and roll of participants in real-time. Moreover, the deviFigce is able to perform the correction through localized vibrotactile stimuli when different abnormal posture states are detected and time-lasting conditions are satisfied. Although individual results vary, the temporal plots clearly demonstrate that all participants returned to the normal state (green area) in several seconds, i.e., 100% successful corrections. This validated the effectiveness of using the e-skin device for neck posture monitoring and vibrotactile correction.

Furthermore, three participants from the Book scenario triggered haptic correction in the pitch direction more than 20 times (B1, 20; B3, 24; and B4, 23). This means that in less than 1 min, those participants would enter the abnormal pitch neck posture at least once. This phenomenon is not observed in the scenarios of playing smartphones and dealing with office files. The depicted results in Fig. 4h further support this conclusion. The mean number of vibrotactile corrections is 4.5, 19.5, and 6.5 for the pitch and 1.0, 3.0, and 2.0 for the roll in the smartphone, book, and computer scenarios, respectively. Error bars indicate standard deviation. In addition, the correction frequency is 0.28, 1.11, and 0.43 per minute for the smartphone, book, and computer scenarios, respectively. The correction frequency of reading comprehension is more than twofold that of playing smartphones and using computers, while playing smartphones demonstrates the lowest correction frequency. We propose that these results logically and reasonably demonstrate the device’s performance during daily human activities. They may further support the proper functioning and performance of the sensing and vibrotactile-correcting capabilities of the e-skin device (as part of its rational design).

### Interview and comments

During our interviews with the participants about user experience, all twelve participants provided positive comments about neck posture sensing and correction of the e-skin device. For example, Participant B2 mentioned that “the e-skin device is good at monitoring and keeping me in a good posture condition of sitting up straight and looking ahead”. Moreover, ten participants commented that the e-skin device is friendly in its form factor and lightweight.

Additionally, five participants from book reading and computer-using scenarios mentioned that the vibrotactile correction strategy definitively helped them perform the correction process by cueing the correction direction when they were highly focused on their specific tasks, such as B1 in the situation of reading book with writing answers on a piece of A4 paper and C1 in the process of copy-typing text on a computer. This implies the suitability for the intuitive cuing of the correction directions for neck postures when the participants are immersed in high-concentration tasks, especially in situations such as book readings and computer office tasks.

## Conclusion

This paper presented an untethered and skin-integrated device with an e-skin form factor. The device is capable of sensing and providing vibrotactile feedback on the neck of the cervical spine to monitor and haptically correct the neck posture. The proposed multilayer structure integrated all flexible electronic circuits and components into a compact skin space for the form factor of an e-skin device. This design scheme was able to make the device untethered, compliant, and skin-conformal when attaching to the neck skin. Moreover, a hollow-structure-based attachment method was proposed for stably attaching e-skin to human neck skin. This method can avoid the position shift of the e-skin device, ensuring the reliability of posture sensing and localized vibrotactile rendering during the movement of neck flexion and lateral bending.

We also evaluated the e-skin device and validated its sensing precision, mechanical properties for skin-conformal compliance, stickiness, and effectiveness in following the change of different neck postures of neck flexion and lateral bending and enabling perceptual isolation of localized vibrotactile discrimination. We conducted a user study that further verified the favorable performance of the soft e-skin device in sensing the posture of participants and performing vibrotactile corrections in situations of using smartphones, reading books, and processing computer files. Thus, the e-skin device might create opportunities for cervical spondylosis prevention and rehabilitation.

However, using a square-shaped e-skin, the skin-conformal compliance for the lateral bending would not be as suitable as the bending for flexion. This means that the posture estimation for the lateral bending could be impacted. Nevertheless, as proven in the user study, flexion abnormities are more frequent in representative human life activities. In addition, a thinner e-skin design can contribute to compliance to follow the lateral bending of users, which is one of our following goals to optimize the device. Furthermore, posture estimation of the IMU-based e-skin device could be impacted by the abnormal posture of the waist (lumbar vertebra posture) and not just the neck posture. This could possibly be optimized through algorithm design by using another e-skin device to monitor the waist posture.

## Discussion

The evaluation of the e-skin device validated its sensing precision, mechanical properties for skin-conformal compliance, stickiness, effectiveness in following the change of different neck postures of neck flexion and lateral bending, and the perceptual isolation of localized vibrotactile discrimination. The user study proved the good performance of the soft e-skin device in sensing the posture of participants and performing vibrotactile corrections. It demonstrates the potential of the proposed compact e-skin to be attached to the neck in study, office, and entertainment situations, which might be quite competitive compared to other neck posture correction devices using rigid designs and audio-visual feedback, because the e-skin form factor device is soft, skin-conformal, untethered, intact, and lightweight, which means it is more user-friendly.

Moreover, we used a digital sound level meter (ID: TES-1359A, Taiwan, accuracy: ±1.5 dB) to measure the sound level at a position 1.4 m away from the e-skin. It shows a value of 36.8 dB (lab environment sound level: 35.7 dB). The measured value close to the e-skin device is 47.2 dB. As interviewed, vibrotactile actuators encapsulated by silicone and attached to human skin cause faint sounds, causing negligible sound disturbance for other people. In the future, using smaller-sized and low-noise vibrotactile actuators is worthwhile to further decrease the noise of e-skin devices. Meanwhile, the e-skin device directionally stimulates the user’s neck skin for correction to the right posture range, and it might not stop users from continuing to work, such as reading books, office events, and cellphone use. A suddenly appearing window (a pop-up window) for providing visual correction cues on screens of mobile phones and computers may interrupt the user’s current work.

In addition, the user study demonstrates that participants in the scenario of book reading comprehension more commonly enter the abnormal neck posture and frequently activate the vibrotactile correction process (1.11/min) compared with using computers (0.43/min) and smartphones (0.28/min). This can be attributed to the following reasons. Reading book articles and doing the corresponding comprehensions need relatively more attention. During this process, participants write their answers on an A4 paper placed on the desk next to the book, which could be the reason that leads to more neck posture abnormalities. When participants use smartphones, as observed, they sometimes just pick up the smartphone instead of placing it close to the desk. For the computer-use scenario, the relatively large sizes of vertically placed computer screens in front of the participants can contribute to the smaller number of neck posture abnormalities. Unlike using smartphones, the participants needed to look at the unmovable computer screen with the head relatively raised.

The danger state was entered much fewer times than the warning state during the 20-min user study test. This could be due to the following reasons. First, the danger state of neck posture is, in fact, relatively severe (see Fig. 3b, c). Second, the 20 min of the test may not be a real long-term fixed posture in sedentary compared to hours of study or office work. Finally, the participants may subconsciously avoid severe neck posture abnormalities because of the experimental attendance and the subconsciousness of the neck-mounted device of e-skin. On the other hand, in the 20-min test, only two correction events were triggered by the danger state, but the two correction events were from the scenario of book reading (B1, beta, at 1120 s, 12.7°, and B4, alpha, at 10 s, 32.7°). This should probably draw more attention to the cervical spine health of students. Moreover, the correction frequency of reading comprehension (Book scenario, 1.11/min) was more than twofold that of playing smartphones (0.28/min) and using computers (0.43/min). Using the technique of the proposed e-skin form factor device might help students or other people relieve cervical spine problems.

In fact, to the best of our knowledge, for an attachable/wearable skin-integrated device, there have not been any reports that support repeated attaching and detaching without some necessary requirements to clean the contact surface. This is consistent with our experiments, which manifest that the effect of e-skin adhesion degenerates with repeated attachment times due to adhesive material loss and dust/perspiration accumulation.

Therefore, in our study, we adopted a scheme of attaching by using replaceable double-sided medical tapes that can be replaced to guarantee adhesion. In the future, for the convenience of users, suction-cup-based adhesion or a sticking solution that supports repeated washing can be investigated and studied to optimize the adhesion effect of skin-integrated devices.

Regarding the classification method of the severity level of human neck posture, for different individual neck postures, in the future, e-skin devices can be programmed and support individual settings according to clinical suggestions using a software app.

As people typically work or study for a whole morning (9:00–12:00) and/or a whole afternoon (14:00–17:00) with breaks for eating and resting at noon, we attached two e-skin devices to two participants for an entire day, except during the noon break. Subsequently, the two participants were interviewed and reported no irritations or discomfort in the skin area where the devices were attached. The experimenter also observed nothing unusual in that skin area. Furthermore, since we used skin-safe silicone (Ecoflex^TM^ 00–33) to manufacture the e-skin device and double-sided medical adhesive tape to attach it to the neck, we believe the e-skin device is gentle on human skin.

Additionally, after a full day of attachment except noon break, we still used a digital level (GIM60 Professional) to measure the angle pitch and roll of the e-skin using the same setup and process as described earlier. The root mean squared errors are ${{{\alpha }}}_{{{RMSE}}}={{1.28}}^\circ$ and ${{{\beta }}}_{{{RMSE}}}={{1.15}}^\circ$, respectively, compared to the previously tested values of ${{{\alpha }}}_{{{RMSE}}}={{1.37}}^\circ$ and ${{{\beta }}}_{{{RMSE}}}={{1.14}}^\circ$, respectively. Therefore, we might conclude that the e-skin sensing precision remains consistent.

To effectively reduce the device thickness, because the maximal thickness of the electronic component is the actuator (9 mm), using a smaller vibrotactile actuator is worthwhile to decrease the overall thickness of the e-skin device. Comprehensive optimization of device selection for other electronic chips and components can further reduce the average thickness of the device.

## Materials and methods

### Fabrication of the e-skin device

As demonstrated in Fig. 1c–f, the architecture, structures, user scene, and details of the e-skin device are shown. Figure 1c displays the 2D ($x$-$y$) illustrations of the silicone-based e-skin, which encapsulates the flexible printed circuit film (FPC film), power battery, and four direction-coding actuators. The adopted flexible FPC film (polyimide/PI) allows for printing all circuits, i.e., chips and flexible connecting copper (Cu) traces, while supporting the overall flexibility and compliance of the integrated e-skin device. These traces interconnect the collection of small, chip-scale integrated circuit components and passive elements, including a microcontroller, sensor, vibration drivers, 2.4 GHz wireless radio frequency microstrip antenna module, resistors, capacitors, and low dropout power regulator, thus controlling the whole sensing and driving system. The accelerometer sensor, with its built-in coordinates ${o}_{s}$-${xyz}$ (as depicted in Fig. 1c), is orthogonally surface-mounted and aligned with the vertical central axis of the FPC film.

The four actuators surrounding the FPC film are implemented as actuator 1 $\to$ 3 (i.e., up $\to$ down) and actuator 2 $\to$4 (i.e., left $\to$ right) configured perpendicularly. The thin, soft piece of silicone material cast between the actuators and the FPC film is used to isolate the four vibrotactile stimuli while simultaneously encapsulating all FPC-mounted electronic components to form an intact e-skin form factor. Filamentary serpentine enameled copper wires are used to electrically connect the four actuators and their drivers, allowing for the stretchability of the silicone material between the actuators and the PFC film. In the scenario depicted in Fig. 1d, with the world coordinates ${o}_{w}$-${XYZ}$, the user is working with office files, inclining her neck forward at a flexion pitch angle $\alpha$ and bending toward the right shoulder at a lateral roll angle $\beta$ while being monitored by the e-skin device.

The entire electronic system is powered with an embedded 3.7 V lithium battery. The total mass of the skin piece is 49.5 g, and a prototype is shown in Fig. 1g. For further information on the parameters of circuits and components, please refer to supplementary material S1.

### Neck posture estimation algorithm

The built-in coordinate ${o}_{s}$-${xyz}$ in the accelerometer sensor (see Fig. 1c) is used as the base coordinate for calculating the real-time neck posture data of pitch and roll ${[\alpha ,\beta ]}^{T}$. The temporal neck posture ${\boldsymbol{f}}\left(t\right){=[\alpha ,\beta ]}^{T}$ is calculated by

1 $$\begin{array}{ll}f(t)=\\{sign}\left(1\right)\cdot \left[\begin{array}{c}\left|\frac{\pi }{2}-{\rm{arctan}} \left({x}_{g}\left(t\right)\cdot \left[{y}_{g}^{2}\left(t\right)+{z}_{g}^{2}\left(t\right)\right]^{-\frac{1}{2}}\right)-{\alpha }_{0}\right|\\ \left|{\rm{arctan}} \left({y}_{g}\left(t\right)\cdot \left[{x}_{g}^{2}\left(t\right)+{z}_{g}^{2}\left(t\right)\right]^{-\frac{1}{2}}\right)\right|\end{array}\right]\end{array}$$

where ${\boldsymbol{a}}=[{x}_{g}$, ${y}_{g}$, and ${{z}_{g}]}^{T}$ indicate the gravity components at the three axes ${o}_{s}-{xyz}$, and $\left|{\boldsymbol{a}}\right|={({x}_{g}^{2}+{y}_{g}^{2}+{z}_{g}^{2})}^{-\frac{1}{2}}=1{\rm{g}}$. ${sign}\left(1\right)=-1$ when ${-\alpha }_{0}\, < \,\alpha \left(t\right) \,<\, 0$ or $-\frac{\pi }{2} < \beta \left(t\right) < 0$, and ${sign}\left(1\right)=1$ when $0 \,<\, \alpha \left(t\right) \,<\, {-\alpha }_{0}+\frac{\pi }{2}$ or $0 \,< \,\beta \left(t\right) \,<\, \frac{\pi }{2}$. ${\alpha }_{0}$ is a zero-calibration value for pitch. As depicted in Fig. 1a, the zero-point angle for pitch is not aligned with the vertically central axis, as the human neck has a natural neck posture curvature of flexion ${\alpha }_{0}$. Lateral bending can result in zero with respect to the body central axis when sitting up straight.

However, raw temporal neck posture data ${[\alpha ,\beta ]}^{T}$ contain noise and may fail the neck posture estimation. In addition, although soft silicone-based e-skin and the set-value of 160 Hz for vibrotactile stimuli^43^ may contribute to user stimulus-location discrimination, actuator vibrations and associated clutter noise can also be sensed by the sensor, leading to posture estimation failure. To address this problem, a Butterworth lowpass filter with a cutoff frequency of 0.5 Hz^14^ is applied.

Specifically, the signal vectors ${{\boldsymbol{a}}}_{{\boldsymbol{i}}}=[{x}_{{gi}}$, ${y}_{{gi}}$, ${{z}_{{gi}}]}^{T}\epsilon {{\boldsymbol{R}}}^{{\boldsymbol{3}}}$ are obtained from the sensor at time ${t}_{i}$, and a time interval of 1 ms (${t}_{i+1}-{t}_{i}=1{\rm{m}}{\rm{s}}$) is set for sampling and filtering using a first-order Butterworth filter (*n* = 1). Thus, neck posture ${\boldsymbol{c}}={\left[{\alpha }_{c},{\beta }_{c}\right]}^{T}$ can be obtained as follows:

2 $${\boldsymbol{c}}={\boldsymbol{f}}\left[{{\mathcal{F}}}^{-1}\left({\rm{|}}{{\boldsymbol{a}}}_{{in}}\left(j\varOmega \right){\rm{|}}\cdot {\left[{\left(\frac{\varOmega }{{\varOmega }_{0}}\right)}^{2n}+1\right]}^{-1/2}\right)\right]$$

where ${{\boldsymbol{a}}}_{{in}}(j\Omega )$ represents the sampling data ${{\boldsymbol{a}}}_{{\boldsymbol{i}}}$ in the frequency domain, $j={\left(-1\right)}^{1/2}$, ${\Omega }_{0}$ is the $-3{dB}$ cutoff radiant frequency, ${{\mathcal{F}}}^{-1}(* )$ is the Fourier inversion, and ${\boldsymbol{f}}[* ]$ is Eq. (1).

To the best of our knowledge, we have not seen any reports on a unified classification method of severity level of human neck posture in the literature database. Therefore, four of twelve participants were randomly chosen and were asked to perform neck flexion (${{\alpha }}$) and lateral bending (${{\beta }}$) to the extremes when he or she felt uncomfortable with the neck. We found that the maximum value ${{\alpha }}$ is 37.5° and ${{\beta }}$ is 13.5°. Therefore, we chose ${{\alpha }}$ > 30.0° as the danger state and let ${{\alpha }}$ = 15.0° be the dividing line between the warning and normal states. The extension state is decided after users wear the e-skin. Similarly, we chose ${{\beta }}$ > 10.0° as the danger state and let ${\boldsymbol{\alpha }}$ = 5.0° be the dividing line between the warning and normal states. Thus, symbolic functions (3) and (4) are proposed to map the present neck posture ${\boldsymbol{c}}={[{{\boldsymbol{\alpha }}}_{{\boldsymbol{c}}},{{\boldsymbol{\beta }}}_{{\boldsymbol{c}}}]}^{{\boldsymbol{T}}}$ into typical classifications ${{s}}({\boldsymbol{c}},{{{t}}}_{{{l}}})$,

3 $$s({\alpha }_{c},{t}_{l})=\left\{\begin{array}{c}E,-{\alpha }_{0}\le {\alpha }_{c} < 0,{t}_{l}\ge 10s\\ N,0\le {\alpha }_{c} < \frac{\pi }{12},{t}_{l}\ge 0\\ W,\frac{\pi }{12}\le {\alpha }_{c} < \frac{\pi }{6},{t}_{l}\ge 10s\\ D,\frac{\pi }{6}\le {\alpha }_{c} < \frac{\pi }{2}-{\alpha }_{0},{t}_{l}\ge 10s\end{array}\right.$$

4 $$s\left({\beta }_{c},{t}_{l}\right)=\left\{\begin{array}{c}N,-\frac{\pi }{36}\le {\beta }_{c}\le \frac{\pi }{36},{t}_{l}\ge 0\\ W,-\frac{\pi }{18}\le {\beta }_{c} < -\frac{\pi }{36}{or}\frac{\pi }{36} < {\beta }_{c} < \frac{\pi }{18},\\ {t}_{l}\ge 10s\\ D,-\frac{\pi }{4}{ < \beta }_{c} < -\frac{\pi }{18}{or}\frac{\pi }{18}{ < \beta }_{c} < \frac{\pi }{4},\\ {t}_{l}\ge 10s\end{array}\right.$$

where $E,N,{W},$ and ${D}$ indicate that the neck posture of flexion ${\alpha }_{c}$ or lateral bending ${\beta }_{c}$ is in the state of *extension, normal, warning, and danger*, respectively. ${t}_{l}\ge 10s$ indicates that the state lasts 10 s or longer. Lateral bending classifications do not have an *extension* state. Flexion has an asymmetrical posture changing range and can include a posture of head extension related to the healthy posture. For two directions of lateral bending, the posture states of *Warning* and *Danger* are symmetrical.

Based on the classifications of Eqs. (3) and (4) corresponding to flexion and lateral bending, vibrotactile feedback strategies can be proposed.

### Vibrotactile feedback strategy

The linear resonance actuator (LRA) generates a sinusoidal vibration $a\left(t\right)$ perpendicular to the neck skin, as shown by the vibrating direction $m$ in Fig. 1f. The vibration is given by $a\left(t\right)=A\sin (2{\rm{\pi }}{f}_{r}t)$, where ${f}_{r}$ = 160 Hz is the modulated voltage signal of the driver, and $A=3{\mathrm{g}}m/{s}^{2}$ is the maximal vibration amplitude.

The goal of vibrotactile feedback strategies is to prevent users from experiencing abnormal cervical conditions by correcting neck posture conditions from *extension* ($E$), *warning* ($W$), or *danger* ($D$) to *normal* ($N$).

As reported in^44^, the human central nervous system can accurately register the location of a tactile stimulus applied to sites all over the body, and this stimulation easily catches and guides one’s attention in the direction of the cue. For example, a single tap on the right shoulder usually makes a person turn to the right^44^. This natural mapping between body position and egocentric orientation is a great asset for tactile displays that stimulate multiple body sites. Therefore, this advantage prompted us to use four localized vibrotactile stimuli to cue and guide users for neck posture correction. As shown in Fig. 1c, we use actuator 1 (up) to code the direction of neck flexion correction upward (i.e., for raising the head), actuator 3 (down) for correcting neck flexion downward (i.e., for lowering the head), actuator 2 (left) for neck lateral bending toward the left, and actuator 4 (right) for correcting neck lateral bending toward the right. Specifically,

5 $${cor}{rect}\left({\boldsymbol{c}}\right)=\left\{\begin{array}{c}{{downwards}}_{{vib}{{\_}}3},{-\alpha }_{0}\, <\, {\alpha }_{c} \,<\, 0\\ {{upwards}}_{{vib}{{\_}}1},\frac{\pi }{12} \,< \,{\alpha }_{c} \,<\, \frac{\pi }{2}{-\alpha }_{0}\\ {{right}{{\_}}{bending}}_{{vib}{{\_}}4},-\frac{\pi }{4} \,<\, {\beta }_{c} \,<\, -\frac{\pi }{36}\\ {{left}{{\_}}{bending}}_{{vib}{{\_}}2},\frac{\pi }{36} \,<\, {\beta }_{c} \,<\, \frac{\pi }{4}\end{array}\,\right.$$

where ${{downwards}}_{{vib\_}3}$ indicates a correction direction of lowering the head using actuator 3, and similarly for understanding the defined ${{upwards}}_{{vib\_}1}$, ${{right\_bending}}_{{vib\_}4}$, and ${{left\_}{bending}}_{{vib\_}2}$.

Based on the level of abnormal neck postures of *Extension* ($E$), *Warning* ($W$), and *Danger* ($D$) in Eqs. (3) and (4), three kinds of actuating-resting periods $T$ (1 s, 0.5 s, 0.2 s) are used to cue the correction urgency. $T$ = 1 s means the LRA is actuating for 1 s and resting for 1 s, similarly for $T$ = 0.5 s and $T$ = 0.2 s. The shorter the period of vibrating-resting, the more severe the neck posture urgency state^45^. Normal neck posture elicits no vibrotactile feedback.

### Supplementary information

e-skin manufacture details
