# Supplementary material for: A skin-integrated device for neck posture monitoring and correction
Source: Microsyst Nanoeng. 2023 Nov 28;9:150. doi: 10.1038/s41378-023-00613-0 (PMC10682425; doi:10.1038/s41378-023-00613-0)
Supplement: Supplementary file 1 — e-skin manufacture details [file 41378_2023_613_MOESM1_ESM.pdf]

# Supplementary material

## S1. The fabrication process and the parameters of circuits and components

For the manufacturing, the silicone casting process in vertical direction, assemble relationships, and details of the encapsulated e-skin can be seen in Fig. S1. a. The sticky Silicon base (Ecoflex™ 00-33) around 1.2mm thickness is the first layer to be casted using 3D-printed mold, and the four locations of actuators on the Silicone base are designed to be hollow (i.e., the hollow-structure), which is for the actuators (10.0 mm × 22.6 mm × 9.0 mm) being able to attach to the human skin by using the replaceable medical adhesive double-sided tapes.

Electronic components including the accelerometer are soldered onto the specific locations of the Cu-traces printed FPC-film (24.3 mm × 32.7 mm × 0.13 mm). Next, the FPC film, the four actuators, and the power battery, are mounted onto the designed locations of the Silicone base. Then eight threads of filamentary serpentine enamelled-copper-wires (customized wind, with the cross-sectional area 0.035 $mm^2$) are separately soldered to electrically connect the four actuators and their drive-chips (DRV8833, TI) on the FPC (same process to connect the 19.5 mm × 10 mm × 3.2 mm battery).

After that, because the thickness of actuators (9.0 mm) compared to other components (thickest chip: 1.8 mm, battery: 3.2 mm) is rather thicker, the Silicone cover is designed to be casted in two steps: casting the silicone cover for the area excluding the actuators (for coating FPC) and the area for wrapping actuators. And the actuators being totally wrapped by the finally casted ~0.2mm thickness Silicone cover layer would not drop out from the hollow of the Silicone base, as the shown inset in Fig. S1. b, cross-sectioned by $p$ in plane $YOZ$, which shows the cured silicone e-skin thickness parameters. The layered silicone casting scheme from the base to the cover resulted in e-skin thickness discrepancies between the area of actuators (~9.1 mm) and the area of no-actuators (~3.1 mm).

In the end, the Silicone cover curing with the Silicone base results in a completely encapsulated e-skin (71.0 mm × 66.0 mm) which is able to coat and stick to the neck skin providing the neck posture sensing and vibrotactile feedback.

The triaxial accelerometer is ADXL350 (Analog Devices Inc., USA) with high resolution (13-bit) and it has an output data rate up to 3200Hz. The Linear Resonance Actuator (LRA, AFT14, Alps, Japan) is adopted owing to its capability of working at resonance frequency $f_r = 160$ Hz (3g $m/s^2$ at 3 V supplied) which is in the range of human most sensitive band of vibration. The microcontroller (MCU, STM32F030-K6T6, STMicroelectronics) reads data from accelerometer, and resolves the user neck posture using corresponding algorithms. If the algorithm infers an abnormal neck posture of users, corresponding designed strategies of vibrotactile stimuli would be performed for haptically cuing and correcting. An integrated 2.4G wireless UART module (Bluetooth protocol, CH9143, WCH Semiconductor) in the e-skin is for sending the user neck posture data and temporal information to a laptop for storage and analysis.

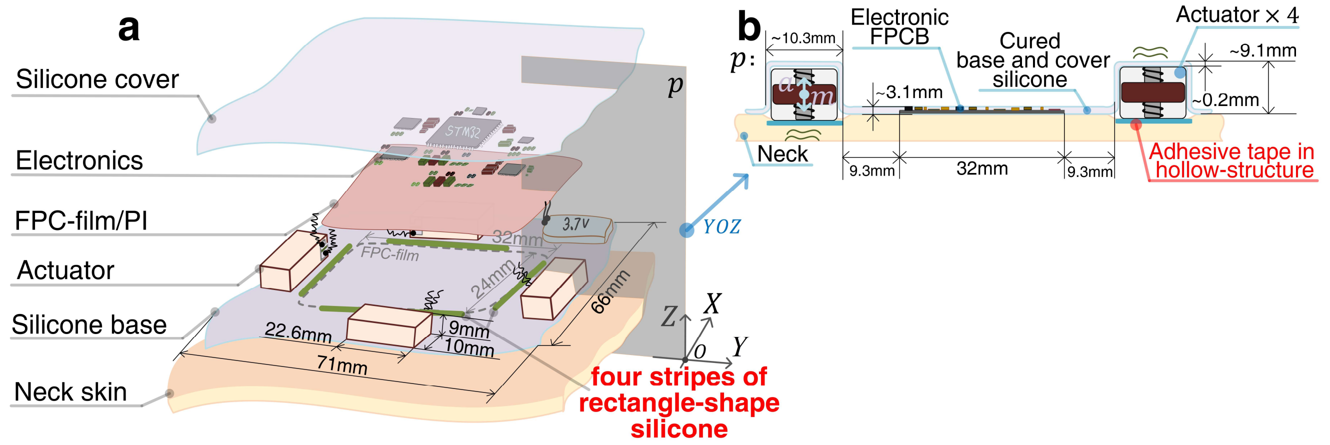


**Fig. S1. The fabrication process of the e-skin device.** **a** shows the details on the encapsulation and fabrication process. **b** shows the cross-sectioned view of the encapsulated e-skin.
